# Genetic structure and geographical distribution of *Bithynia siamensis* sensu lato from Khong and Mounlapamok districts, Champasak Province, Laos

**DOI:** 10.1186/s41182-025-00720-w

**Published:** 2025-04-02

**Authors:** Naruemon Bunchom, Weerachai Saijuntha, Virasack Bounavong, Bounmixay Pakouakeu, Parita Hansana, Pheovaly Soundala, Chavanut Jaroenchaiwattanachote, Takeshi Agatsuma, Marcello Otake Sato, Philippe Buchy, Moritoshi Iwagami

**Affiliations:** 1 Department of Tropical Medicine and Malaria, Research Institute, National Center for Global Health and Medicine, Tokyo, Japan; 2 SATREPS Project for Parasitic Diseases (JICA/AMED), Vientiane, Laos; 3 Faculty of Medicine, and Biomedical Science Research Unit, Mahasarakham University, Mahasarakham, Thailand; 4 Center of Excellence in Biodiversity Research, Mahasarakham University, Mahasarakham, Thailand; 5 Center of Malariology, Parasitology and Entomology, Ministry of Health, Vientiane, Laos; 6 Lao Tropical and Public Health Institute, Ministry of Health, Vientiane, Laos; 7 Institut Pasteur du Laos, Ministry of Health, Vientiane, Laos; 8 Department of Environmental Health Sciences, Kochi Medical School, Nankoku, Kochi Japan; 9 Faculty of Medical Technology, Division of Global Environment Parasitology, Niigata University of Pharmacy and Medical and Life Sciences, Niigata, Japan

**Keywords:** Cholangiocarcinoma, Opisthorchiasis, Genetic variation, Gene flow, Migration

## Abstract

**Background:**

*Bithynia* spp., a key intermediate host of *Opisthorchis viverrini*, is widely distributed in the lower Mekong sub-region, where opisthorchiasis remains a major public health concern. Understanding the genetic diversity and population structure of these snails is crucial for disease control. *Bithynia siamensis* sensu lato has been classified into three genetic lineages (I–III) based on cytochrome *c* oxidase subunit 1 (*cox1*) and 16S ribosomal RNA (16S rRNA) sequence analysis. This study focuses on Champasak Province, Laos, a highly endemic area of opisthorchiasis with limited genetic data on *Bithynia* spp.

**Methods:**

*Bithynia* snails were collected from 12 villages in Khong and Mounlapamok districts, Champasak Province, Laos, between February and August 2024. To compare with previous reports, a total of 246 and 139 samples were analyzed using *cox1* and 16S rRNA markers, respectively. Genetic diversity, genetic differentiation, and genetic structure were assessed based on these markers. Haplotype networks were constructed based on *cox1* and 16S RNA sequences to elucidate the genetic lineage of these samples.

**Results:**

In the present study, only *Bithynia siamensis goniomphalos* was identified, while *B. s. siamensis* and *B. funiculata* were not found. Our findings revealed that both *cox1* and 16S rRNA sequences exhibited high haplotype diversity among populations but relatively low nucleotide diversity. Two lineages of *B. s. goniomphalos* (lineages II and III) were detected in the studied areas, exhibiting significant genetic structuring among groups of snail populations from different villages in each lineage. Notably, lineage II was identified in Laos for the first time. The distribution of lineage II was observed near the southern border of Laos and Cambodia.

**Conclusions:**

This study is the first to use DNA analysis to investigate *Bithynia* spp. in opisthorchiasis-endemic areas of Champasak Province, where *B. s. goniomphalos* lineages II and III were detected, but lineage I was not found. Our finding suggested that geographic or environmental factors influence the distribution of specific *Bithynia* lineages in this region. Many *O. viverrini* endemic areas in Southeast Asia still lack genetic data on *Bithynia* snails which could provide valuable insights into the transmission dynamics of opisthorchiasis. Therefore, further investigations should be conducted in these areas using *cox1* and 16S rRNA sequences for comparison with previous studies.

## Background

The Mekong River is one of Asia’s longest transboundary rivers, spanning 4350 km and covering a floodplain area of 810,000 km^2^. It flows through South China, Myanmar, Thailand, Laos, Cambodia, and Vietnam, forming the upper and lower Mekong Basins. The lower Mekong sub-region encompasses most of Laos and Cambodia, the northern and northeast parts of Thailand, and the Mekong Delta and Central Highlands of Vietnam. This geographically diverse region includes tropical, subtropical, temperate, and alpine ecoregions, with mountain peaks generally below 3048 m (Mekong River Commission; www.mrcmekong.org; accessed on 19 Nov 2024).

The World Health Organization (WHO) has identified food-borne diseases as a global public health priority (World Health Organization; www.who.int/activities/estimating-the-burden-of-foodborne-diseases; accessed on 19 Nov 2024). Several diseases are transmitted by snail intermediate hosts. *Bithynia* spp., a freshwater bithyniid snail, is widely distributed across the lower Mekong sub-region, including Thailand, Cambodia, Myanmar, Laos, and southern Vietnam [1–7]. It serves as the first intermediate host of the liver fluke *Opisthorchis viverrini* (Poirier, 1886), a parasite associated with cholangiocarcinoma, a significant public health concern in Southeast Asia. In Laos and Thailand, at least 10 million people are suffering from opisthorchiasis, with approximately 67 million people at risk of infection [8, 9].

The life cycle of *O. viverrini* is complex, requiring three hosts, namely, freshwater *Bithynia* snails as the first intermediate host; freshwater cyprinid fish (at least 40 species within 18 genera) as the second intermediate hosts [10]; and humans as the definitive hosts. Several mammals, including cats, dogs, and pigs, served as reservoir hosts [11]. Three taxa of *Bithynia* have been reported in mainland Southeast Asia, namely, *Bithynia funiculata* (Walker, 1927), *Bithynia siamensis siamensis* (Morelet, 1866), and *Bithynia siamensis goniomphalos* (Morelet, 1866). Among these, *B. s. goniomphalos* is the most widespread species, occurring across northern, central, northeastern, and western Thailand, as well as in Laos, Cambodia, and southern Vietnam [4–7]. These snails thrive in paddy fields and croplands, where environmental factors, such as altitude, temperature, soil, salinity, and pH influence their distribution [12, 13]. In addition, these snails also serve as intermediate hosts for other medically important trematodes, such as echinostomes and non-human schistosomes [14, 15].

Genetic diversity within host species is a crucial factor in understanding disease epidemiology. Population connectivity, which links spatially separated populations, involves dispersal dynamics and the comparison of genetic diversity patterns [16]. Furthermore, genetic markers, such as DNA barcoding are essential for resolving uncertainties in morphological taxonomy, particularly when identifying cryptic species with similar morphology but distinct genetic identities [4–6]. In addition, nucleotide polymorphisms in nuclear DNA like the intron regions of arginine kinase have been used to differentiate *Bithynia* spp. in Thailand [7]. Anthropogenic factors, such as dam construction and water resource development, can disrupt the natural flow of the Mekong River, hindering the dispersal of snail hosts and promoting hybridization events [17].

Previous studies have identified three genetically distinct lineages (I–III) of *B. s. goniomphalos*. In Thailand, lineage I was the most prevalent, occurring across the northern, central, northeastern, and western regions [4–6]. In Cambodia, lineage II was predominant. In Laos, lineage I was found in the northern and central regions, while lineage III occurred in the central and southern regions [2]. Understanding the genetic structure and diversity of *Bithynia* spp. is essential for elucidating the co-evolutionary dynamics of host–parasite systems and informing the development of effective long-term prevention and control strategies. For example, as the allele spreads through the population, a greater proportion of snails will be resistant to the parasite, meaning fewer snails are susceptible to infection. At the same time, the adaptive dynamic of host–parasite is shaped by a continuous cycle of adaptation and counter-adaptation [18].

Champasak Province, Laos, has a high prevalence of *O. viverrini* infection and genetic data on *Bithynia* snails is absent. In addition, the Khong and Mounlapamok districts are situated near the borders of Thailand, Cambodia, and Laos, making them a key location for studying cross-border transmission dynamics and interactions between neighboring countries. Thus, in this cross-sectional study, we aim to investigate the genetic diversity, population structure, geographical distribution, and phylogenetic relationships of *B. siamensis* sensu lato in various localities of Champasak Province, Laos. The mitochondrial cytochrome *c* oxidase subunit 1 (*cox1*) and 16S ribosomal RNA (16S rRNA) were used as genetic markers, which have been proven to be DNA barcoding. This study will provide the first genetic data on these snails in the recently studied areas, contributing to a better understanding of their species status and distribution.

## Methods

### Sample collection

**Fig. 1:**
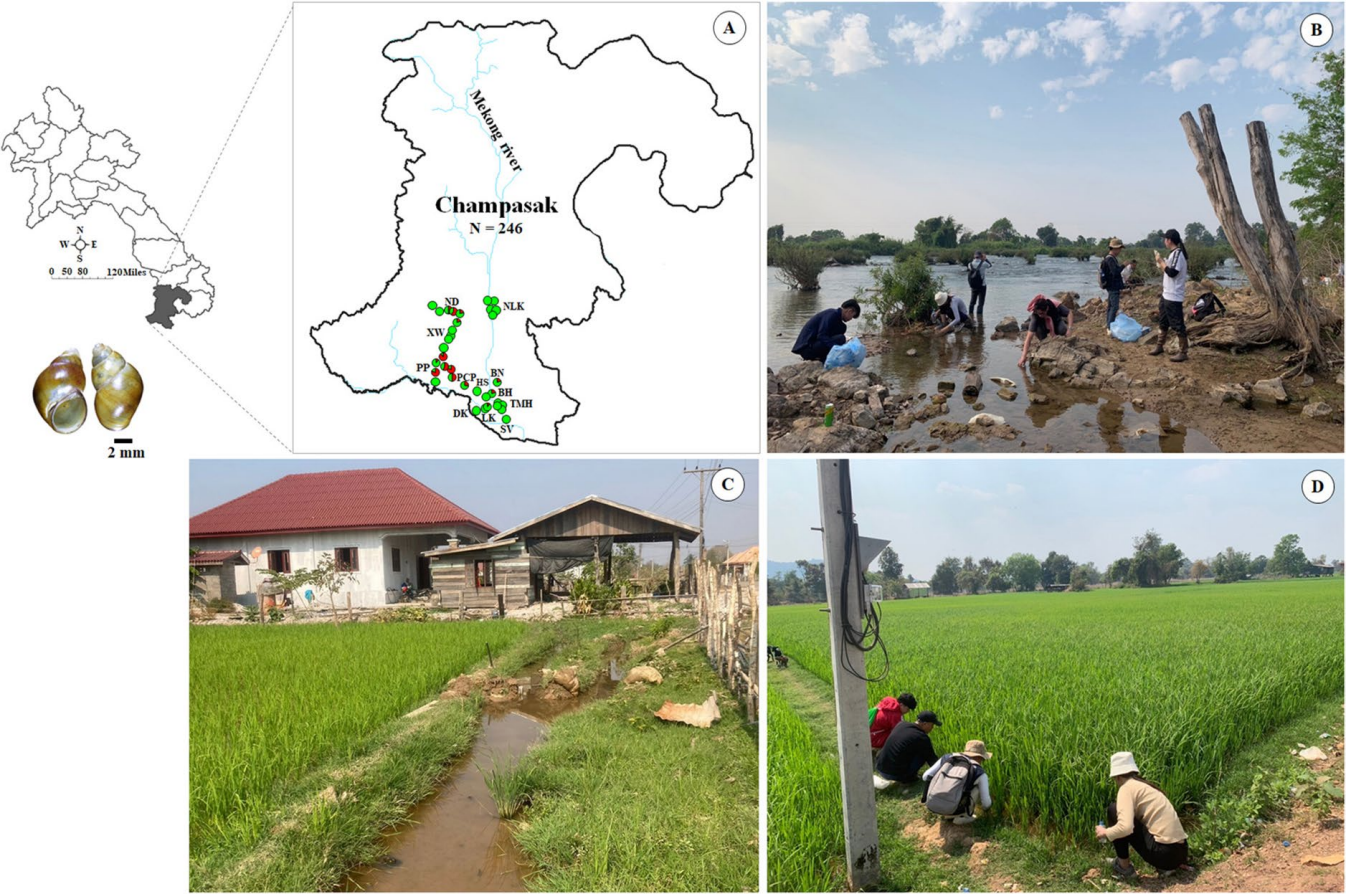
Map of sample collections and natural habitats of *Bithynia siamensis goniomphalos* in 12 villages from Champasak Province in Laos: **A** sampling locations; **B** river near Mekong river; **C** small canal near household; **D** rice field

*Bithynia* spp. samples were collected quarterly in February, May, August, and October 2024 from 35 localities across 12 villages in Champasak Province, southern Laos (Fig. 1 and Table 1). Snails were collected from natural habitats, dried with tissue paper, wrapped in newspaper, and sent to the Institut Pasteur du Laos laboratory. All samples were identified using standard morphological criteria described by Brandt [1], Upatham et al. [2], and Chitramvong [3]. Each individual was placed in hot water at 70 °C for 30–45 s. The head–foot/body was then carefully removed from the shell using forceps, and the head–foot was preserved in an 80% alcohol solution for DNA extraction until further use. The shell and operculum were dried at 70 °C in a hot-air oven overnight and stored in a sample box for morphological study at the Institut Pasteur du Laos laboratory in Vientiane, Laos.

**Table 1:** Localities of *Bithynia siamensis goniomphalos* sample collection from Champasak in Laos

Code	Number	Date		Habitat	Village	District	Latitude	Longitude
HS01	10	Feb 2024		Rice field	Ban Hinsiw	Khong	14.083240	105.828545
BH01	10	Feb 2024		Rice field	Ban Huay	Khong	14.082769	105.839630
BH02	12	Feb 2024		Rice field	Ban Huay	Khong	14.082931	105.839105
BN01	11	Feb 2024		Rice field	Ban Na	Khong	14.104553	105.849084
PCP01	9	Feb 2024		Rice field	Phon Champa	Khong	14.095875	105.775275
SV01	2	Feb 2024		Rice field	Somven-ok	Khong	14.016058	105.898182
PP06	18	Feb 2024		Rice field	Phon Peuy	Khong	14.119862	105.736515
PP07	9	Aug 2024		Rice field	Phon Peuy	Khong	14.125056	105.740306
PP11	7	Aug 2024		Pond	Phon Peuy	Khong	14.113000	105.734556
PP12	9	Aug 2024		Rice field	Phon Peuy	Khong	14.118361	105.734556
PP13	9	Aug 2024		Rice field	Phon Peuy	Khong	14.117750	105.740528
PP14	9	Aug 2024		Rice field	Phon Peuy	Khong	14.116889	105.743389
PP15	6	Aug 2024		Pond	Phon Peuy	Khong	14.115722	105.747278
DK03	5	Feb 2024		Mekong River	Don Khone	Khong	14.040713	105.790748
TMH05	3	May 2024		Rice field	Thamakhep	Khong	14.035059	105.886038
TMH06	6	May 2024		Pond	Thamakhep	Khong	14.033899	105.886494
TMH07	6	May 2024		Rice field	Thamakhep	Khong	14.034484	105.886160
TMH08	5	May 2024		Rice field	Thamakhep	Khong	14.034488	105.886168
LK10	10	May 2024		Rice field	Longkang	Khong	14.042361	105.799500
LK11	5	May 2024		Pond	Longkang	Khong	14.043139	105.798389
XW05	4	May 2024		Pond	Xanwa	Mounlapamok	14.145132	105.744332
XW10	7	May 2024		Rice field	Xanwa	Mounlapamok	14.146250	105.745694
XW11	4	May 2024		Rice field	Xanwa	Mounlapamok	14.146778	105.746639
XW12	2	May 2024		Pond	Xanwa	Mounlapamok	14.147722	105.747194
XW14	5	May 2024		Pond	Xanwa	Mounlapamok	14.150139	105.749611
NLK05	8	May 2024		Pond	Nangloy Kang	Mounlapamok	14.404445	105.849658
NLK07	5	May 2024		Rice field	Nangloy Kang	Mounlapamok	14.430944	105.853611
NLK08	4	May 2024		Rice field	Nangloy Kang	Mounlapamok	14.430694	105.852472
NLK13	9	Aug 2024		Rice field	Nangloy Kang	Mounlapamok	14.403833	105.851361
NLK14	4	Aug 2024		Rice field	Nangloy Kang	Mounlapamok	14.403944	105.853694
ND08	5	May 2024		Pond	Nady	Mounlapamok	14.171639	105.739389
ND10	6	May 2024		Rice field	Nady	Mounlapamok	14.170972	105.741111
ND11	3	May 2024		Rice field	Nady	Mounlapamok	14.169806	105.742722
ND13	10	Aug 2024		Pond	Nady	Mounlapamok	14.165167	105.744361
ND16	9	Aug 2024		Rice field	Nady	Mounlapamok	14.174083	105.736611
**Total**	**246**							

### Molecular analysis

The DNA of each individual was extracted using DNeasy Blood and Tissue Kits (QIAGEN. Co. Ltd., Germany) following the manufacturer’s protocols for molecular study. The *cox1* region was amplified using polymerase chain reaction (PCR) with primers LCO1490/HCO2198 [19], whereas 16 S rRNA amplification was performed with primers 16sar-L/16sbr-H [20]. The GXL premix (Takara Bio. Co. Ltd., Japan) contained 10 µl of premix, 0.4 µl of each primer (10 pM), 7.2 µl of distilled water, 2 µl of DNA template (~ 10 to 20 ng). The PCR conditions used to amplify both the *cox1* and 16S rRNA genes were the same as those described in an earlier paper [6]. The PCR products were checked by electrophoresis on a 2% agarose gel. The amplicons with the expected sizes (approximately 660 and 490 bp for *cox1* and 16S rRNA, respectively) were cut and purified using the FastGene™ Gel/PCR Extraction kit (Nippon Genetics. Co. Ltd., Japan). DNA sequencing was performed using the Applied Biosystems 3130xl Genetic Analyzers (Hitachi. Co. Ltd., Japan).

### Data analyses

The DNA sequences were checked and edited using BioEdit v.7.2.6 [21] (Hall, 1999). All these sequences were aligned using ClustalW (ver. 2, see http://www.clustal.org/clustal2/; Thompson et al. [22]). The *cox1* and 16S rRNA sequences of *Bithynia siamensis goniomphalos* were checked for stop codons and frame-shift mutations and then deposited in GenBank under the accession numbers (PQ848607–PQ848852) and (PQ848853–PQ848991) for the *cox1* and 16S rRNA sequences, respectively. This study included 418 *cox1* DNA sequences of *B. s. goniomphalos*, retrieved from GenBank, which were similar to our sequences: 30 sequences (KY118603–KY18632) were reported by Kulsantiwong et al*.* [4]; 289 sequences (MN399385–MN399673) were reported by Tantrawatpan et al. [5]; and 99 sequences (MW832397–MW832421) were reported by Bunchom et al. [6], respectively. The 96 16S rRNA sequences (MN400112–MN400207) generated by Tantrawatpan et al. [5] and the 52 sequences (MW832397–MW832421) published in GenBank by Bunchom et al. [6] were also included. All of these sequences were included for data analyses, which were multiply aligned for the *cox1* and 16S rRNA using BioEdit version 7.2.6 [21]. The DNA sequences of *cox1* were used to identify the species of *Bithynia* spp. by comparing them with those available in GenBank. To evaluate the level of genetic diversity of these snails from different villages, two genetic diversity indices, haplotype diversity (Hd) and nucleotide diversity () were calculated using the *cox1* and 16S rRNA data with the software DnaSp v.5.10.01 [23]. Pairwise genetic difference (Φ_ST_) and analysis of molecular variance (AMOVA) were calculated in Arlequin v.3.5.2.2 [24]. The phylogenetic tree was constructed through both the neighbor-joining (NJ) and the maximum likelihood (ML) utilizing the *cox1* data which included additional sequences from Thailand, Cambodia, and Laos from GenBank with the MEGA XI program [25]. The general time reversible with among site rate variation sampled from a gamma distribution (GTR + G + I model) [26] was selected for maximum likelihood (ML) with bootstrap support of 1000 replications. Haplotype networks were calculated using a median-joining algorithm [27], with the Network program v.5.0.1.1 (https://www.fluxus-engineering.com/).

## Results

A total of 246 and 139 *Bithynia* snails from 12 villages were analyzed using mitochondrial *cox1* and 16S rRNA gene sequences, respectively. The analysis of 665 bp from the *cox1* sequences identified 58 variable sites, 31 haplotypes (including 14 unique haplotypes), with a haplotype diversity of 0.877 and a nucleotide diversity of 0.019 (Table 2). Similarly, analysis of 498 bp from the 16S rRNA sequences revealed 14 variable sites, 10 haplotypes (including 4 unique haplotypes), with a haplotype diversity of 0.386 and nucleotide diversity of 0.002 (Table 2). Pairwise genetic differentiation (Φ_ST_) among snail populations from the 12 villages—Ban Hinsiw, Ban Huay, Ban Na, Phon Champa, Phon Peuy, Don Khon, Thamakhep, Nangloy Kang, Nady, Longkang, Xanxa, and Somven-ok—revealed significant genetic sub-structuring. The Φ_ST_ values ranged from 0.003 to 0.782 for *cox1* and from 0.010 to 0.975 for 16S rRNA analyses (Table 3).

**Table 2:** Diversity indices of *cox1* and 16S rRNA sequences in the *Bithynia siamensis goniomphalos* populations from 12 villages

Village	*cox1*								16S rRNA							
	N	s	H	Uh	Hd ± SD	± SD	Lineage		N	s	H	Uh	Hd ± SD	± SD	Lineage	
							II	III							II	III
Ban Hinsiw	10	1	5	0	0.556 ± 0.075	0.001 ± 0.000	–	10	10	0	1	0	0.000 ± 0.000	0.000 ± 0.000	–	10
Ban Huay	22	36	7	2	0.801 ± 0.067	0.010 ± 0.004	2	20	20	6	4	1	0.553 ± 0.111	0.002 ± 0.001	2	18
Ban Na	11	34	6	0	0.800 ± 0.114	0.011 ± 0.006	1	10	10	5	3	0	0.644 ± 0.101	0.003 ± 0.001	1	9
Phon Champa	9	32	2	0	0.389 ± 0.164	0.019 ± 0.008	2	7	9	6	2	0	0.389 ± 0.164	0.005 ± 0.002	2	7
Somven-ok	2	3	2	0	0.000 ± 0.000	0.000 ± 0.000	–	2	2	0	1	0	0.000 ± 0.000	0.000 ± 0.000	–	2
Phon Peuy	67	35	8	3	0.728 ± 0.024	0.021 ± 0.002	46	21	8	1	2	1	0.250 ± 0.180	0.001 ± 0.000	8	–
Don Khone	5	3	2	0	0.600 ± 0.175	0.003 ± 0.001	–	5	5	0	1	0	0.000 ± 0.000	0.000 ± 0.000	–	5
Thamakhep	20	5	4	2	0.600 ± 0.077	0.002 ± 0.001	–	20	19	3	4	1	0.380 ± 0.134	0.001 ± 0.000	–	19
Longkang	15	30	3	0	0.514 ± 0.116	0.007 ± 0.005	1	14	15	4	2	0	0.133 ± 0.112	0.001 ± 0.001	1	14
Xanwa	22	33	7	3	0.714 ± 0.085	0.006 ± 0.003	1	21	19	5	3	1	0.205 ± 0.119	0.001 ± 0.001	1	18
Nangloy Kang	30	3	3	0	0.297 ± 0.099	0.001 ± 0.000	–	30	11	0	1	0	0.000 ± 0.000	0.000 ± 0.000	–	11
Nady	33	34	9	4	0.801 ± 0.041	0.017 ± 0.003	7	26	11	5	3	0	0.345 ± 0.172	0.002 ± 0.001	1	10
Total	246	58	31	14	0.877 ± 0.011	0.019 ± 0.001	60	186	139	14	10	4	0.386 ± 0.051	0.002 ± 0.000	16	123

N number of *B. s. goniomphalos* examined, s number of polymorphic sites, H number of haplotypes, Uh unique haplotypes, Hd haplotype

diversity,  nucleotide diversity

**Table 3:** Pairwise genetic differentiation (Φ_ST_) between the populations of *Bithynia siamensis goniomphalos* from 12 different villages by *cox1* (lower triangle) and 16S rDNA (upper triangle) sequences

Village	1	2	3	4	5	6	7	8	9	10	11	12
1. Ban Hinsiw	–	0.026	0.167	0.141	0.973**	0.000	0.000	0.000	0.000	0.000	0.000	0.000
2. Ban Huay	0.056	–	0.000	0.074	0.774**	0.000	0.066	0.034	0.000	0.000	0.011	0.000
3. Ban Na	0.208**	0.003	–	0.087	0.794**	0.063	0.184**	0.181*	0.053	0.100	0.123	0.000
4. Phon Champa	0.161*	0.037	0.061	–	0.674**	0.034	0.187	0.156	0.038	0.091	0.123	0.000
5. Somven-ok	0.688	0.000	0.000	0.000	–	0.947*	0.000	0.000	0.000	0.000	0.000	0.000
6. Phon Peuy	0.519**	0.452**	0.434**	0.362*	0.435	–	0.962**	0.916**	0.887**	0.888**	0.975**	0.836**
7. Don Khone	0.656**	0.128*	0.070	0.124	0.134	0.495**	–	0.000	0.000	0.000	0.000	0.000
8. Thamakhep	0.782**	0.353**	0.368**	0.425**	0.303	0.566**	0.593**	–	0.014	0.012	0.000	0.010
9. Longkang	0.082*	0.000	0.042	0.057	0.033	0.462**	0.125	0.483**	–	0.000	0.000	0.000
10. Xanwa	0.148**	0.016	0.022	0.115*	0.000	0.494**	0.171	0.466**	0.017	–	0.000	0.000
11. Nangloy Kang	0.730**	0.328**	0.324**	0.438**	0.572*	0.587**	0.094	0.730**	0.314**	0.349**	–	0.000
12. Nady	0.147*	0.041	0.007	0.031	0.000	0.333*	0.112*	0.287**	0.058	0.057	0.269**	–

*P* value calculated at a confidence level of 95% * < 0.05, ** < 0.001

**Fig. 2:**
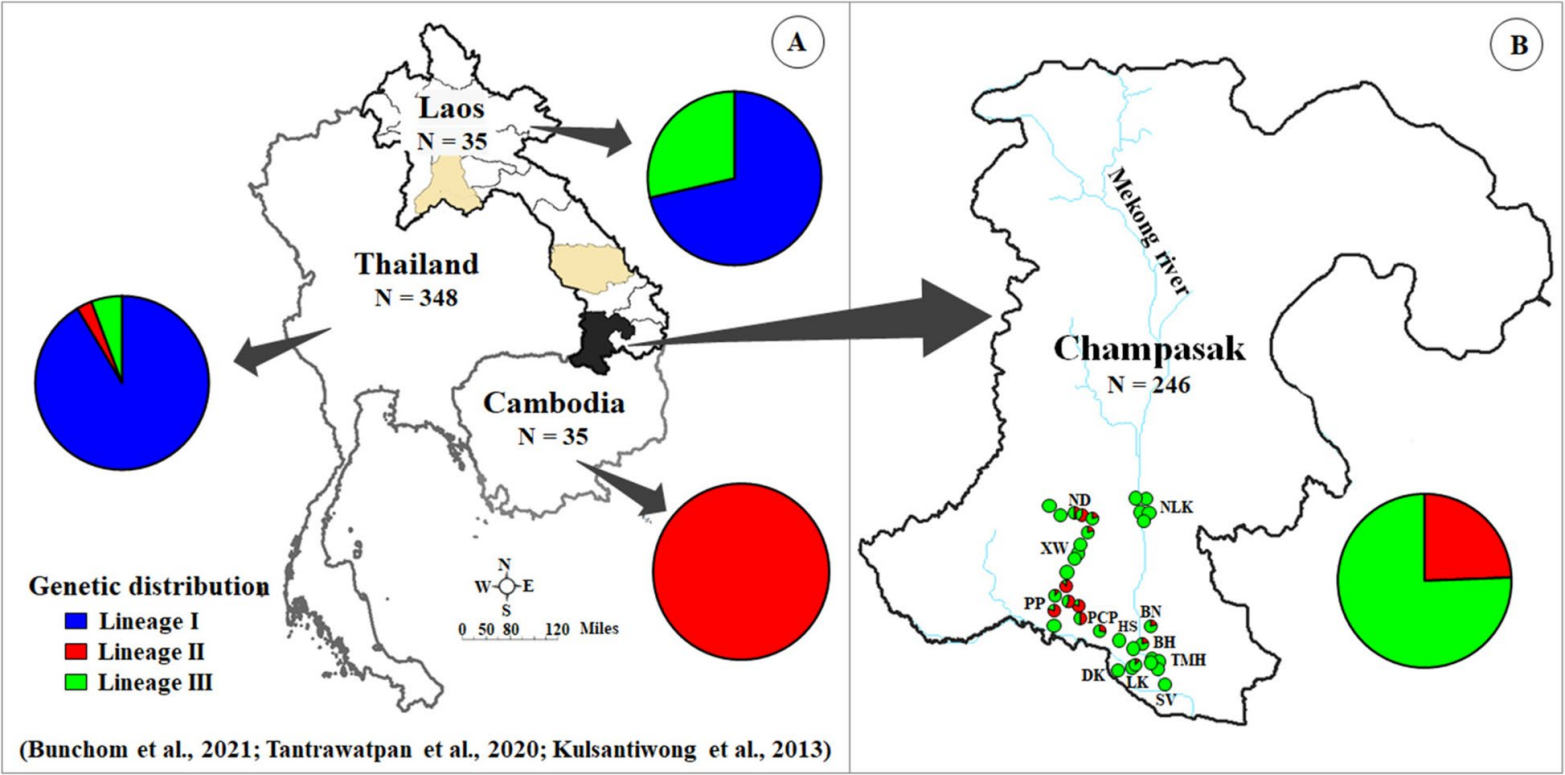
Genetic structure and geographical distribution of *Bithynia siamensis goniomphalos* using *cox1* sequences in Thailand, Cambodia, and Laos: **A** distribution of *B. s. goniomphalos*: data obtained from GenBank; **B** distribution of *B. s. goniomphalos*: present study

The genetic structure and geographical distribution of *Bithynia siamensis goniomphalos* based on *cox1* sequences revealed distinct lineage patterns across Southeast Asia. In Thailand, lineage I was the most common and widely distributed in the northern, central, northeastern, and western regions. In Laos, lineage I was found from the northern to central regions, and lineage II was identified from the southern region, while lineage III was found from the central to southern regions. In Cambodia, only lineage II was detected (Fig. 2).

**Fig. 3:**
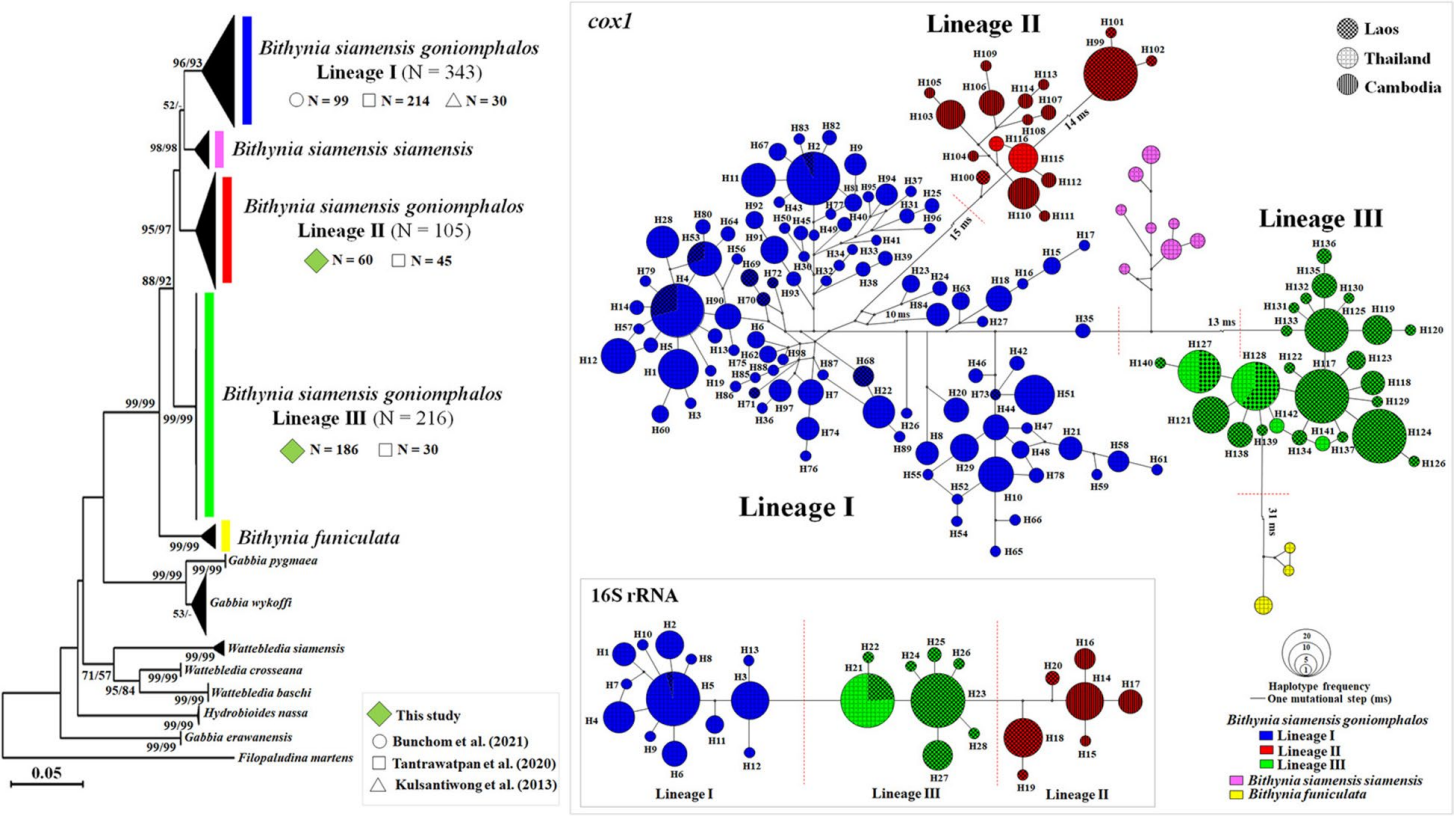
Phylogenetic tree of the bithyniids based on *cox1* sequences in Thailand, Cambodia, and Laos. Neighbor-joining (NJ) and maximum likelihood (ML) with bootstrap support of 1000 replications were presented near branches. Median-joining networks (MJNs) based on *cox1* and 16S rRNA of *Bithynia siamensis goniomphalos* was classified into three (I–III) genetic lineages

A comparison of 606 bp from 664 *cox1* sequences (246 obtained in this study and 418 retrieved from GenBank) revealed 145 variable positions, which were classified into 142 haplotypes (H1–H142) (Tables 2 and 4). The phylogenetic tree and Median-Joining Networks (MJNs) based on *cox1* and 16S rRNA sequences identified three lineages (I–III), whereas samples were classified with *B. s. goniomphalos* lineages II and III (Fig. 3).

**Table 4:**
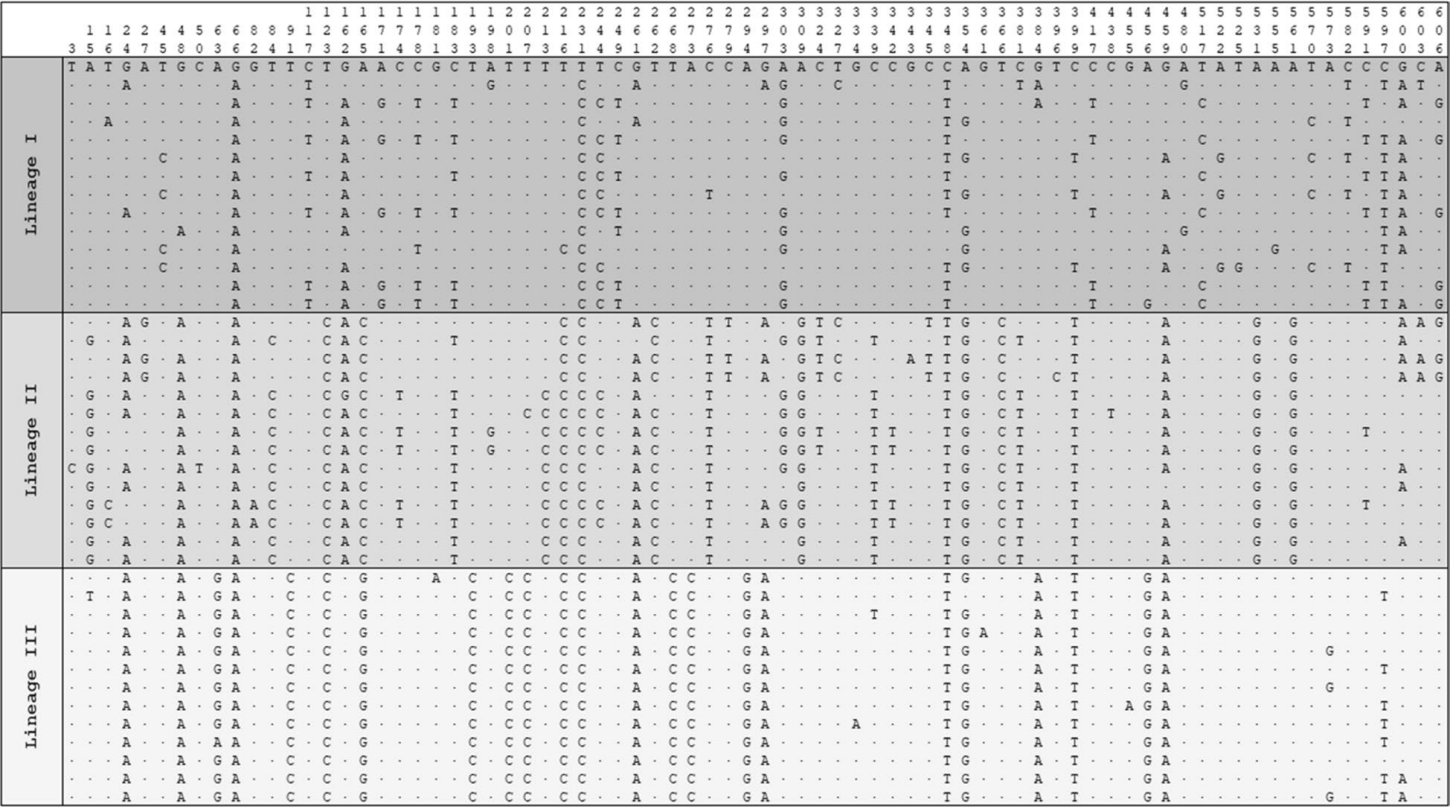
Variable nucleotide positions found in the *cox1* gene comparing of *Bithynia siamensis goniomphalos* between three (I–III) genetic lineages

Since 16S rRNA sequences from other genera of bithyniid snails are not available in GenBank, we were unable to construct a 16S rRNA phylogenetic tree. Therefore, only the *cox1* phylogenetic tree of the bithyniid snails was constructed. The phylogenetic tree revealed that lineage I (*n* = 343) was the largest, comprising 318 sequences from Thailand, and the remaining (25) comprised from Laos. The present study was resolved into lineages II (*n* = 60) and III (*n* = 186). Lineage II comprised 105 sequences from Laos (*n* = 60) obtained in this study plus 10 sequences from Thailand and 35 sequences from Cambodia. Lineage III comprised 216 sequences from Laos (*n* = 196) obtained in this study 186 sequences and the remaining (10) belonged to the former plus 20 sequences from Thailand (Fig. 3).

The *cox1* haplotype network revealed that lineage I (*n* = 343) was the largest, comprising 98 haplotypes from Thailand and Laos. Lineage II consisted of 18 haplotypes from Thailand, Cambodia, and Laos, with haplotype 99 (H99) as a common haplotype in Laos but unshared with other countries. Lineage III included 26 haplotypes from Thailand and Laos, with the majority of samples from Laos (*n* = 201). Within lineage III, haplotype 127 (H127) was shared by 16 samples (three from Thailand and 13 from Laos), and haplotype 128 (H128) was shared by 20 samples (eight from Thailand and 12 from Laos) (Fig. 3).

The 16S rRNA haplotype network also revealed three lineages. Lineage I comprised 13 haplotypes from Thailand and Laos, with haplotype 5 (H5) being shared by 47 samples (45 from Thailand and two from Laos). Lineage II consisted of 7 haplotypes from Thailand and Cambodia, with no haplotypes shared between the two countries. Lineage III included 8 haplotypes from Thailand and Laos, with haplotype 23 (H23) being the largest, represented by 110 samples from Laos (Fig. 3).

The AMOVA was analyzed based on different habitat types and villages (Table 5). Three groups of different habitat types, namely, rice fields, ponds, and Mekong River, were classified. The genetic structures among groups were not significantly different (*F*_CT_ = 0.021, *P* value > 0.05). However, *F*_ST_ was 0.518 (*P* < 0.001), indicating significant genetic differentiation within populations. Most samples were collected from rice fields and ponds, though no significant differentiation was observed among them (Table 5). In addition, the genetic structures among groups from different villages were significant (*F*_CT_ = 0.263, *P* < 0.001), and there was significant genetic differentiation within populations (*F*_ST_ = 0.365, *P* < 0.001) (Table 5).

**Table 5:** Analysis of molecular variance (AMOVA) based on the *cox1* sequences of *Bithynia siamensis goniomphalos* populations

Source of variation	d.f	Ss	Vc	% variation	Fixation index
Group defined by 3 different habitat types [rice field, pond, Mekong River]					
Among groups	2	58.154	0.135	2.11	*F*_CT_ = 0.021
Among populations within groups	32	814.712	3.180	49.73	*F*_SC_ = 0.508**
Within populations	211	649.930	3.080	48.16	*F*_ST_ = 0.518**
Group defined by 7 Villages within lineage II [Ban Huay, Ban Na, Phon Champa, Phon Peuy, Nady, Longkang, Xanxa]					
Among groups	6	36.167	1.257	58.78	*F*_CT_ = 0.222*
Among populations within groups	7	12.733	0.196	9.15	*F*_SC_ = 0.679*
Within populations	46	31.550	0.686	32.07	*F*_ST_ = 0.588**
Group defined by 12 Villages within lineage III [Ban Hinsiw, Ban Huay, Ban Na, Phon Champa, Phon Peuy, Don Khon, Thamakhep, Nangloy Kang, Nady, Longkang, Xanxa, and Somven-ok]					
Among groups	11	102.944	0.512	38.52	*F*_CT_ = 0.177**
Among populations within groups	23	30.839	0.144	10.86	*F*_SC_ = 0.494
Within populations	151	101.674	0.673	50.62	*F*_ST_ = 0.385**
Group defined by 3 different countries within lineage II [Thailand, Cambodia, Laos]					
Among groups	2	387.552	6.377	78.91	*F*_CT_ = 0.660**
Among populations within groups	17	102.915	1.126	13.93	*F*_SC_ = 0.928**
Within populations	85	49.208	0.579	7.16	*F*_ST_ = 0.789**

*P* value calculated at a confidence level of 95% * < 0.05, ** < 0.001

Ss sum of squares, Vc variance components

## Discussion

**Fig. 4:**
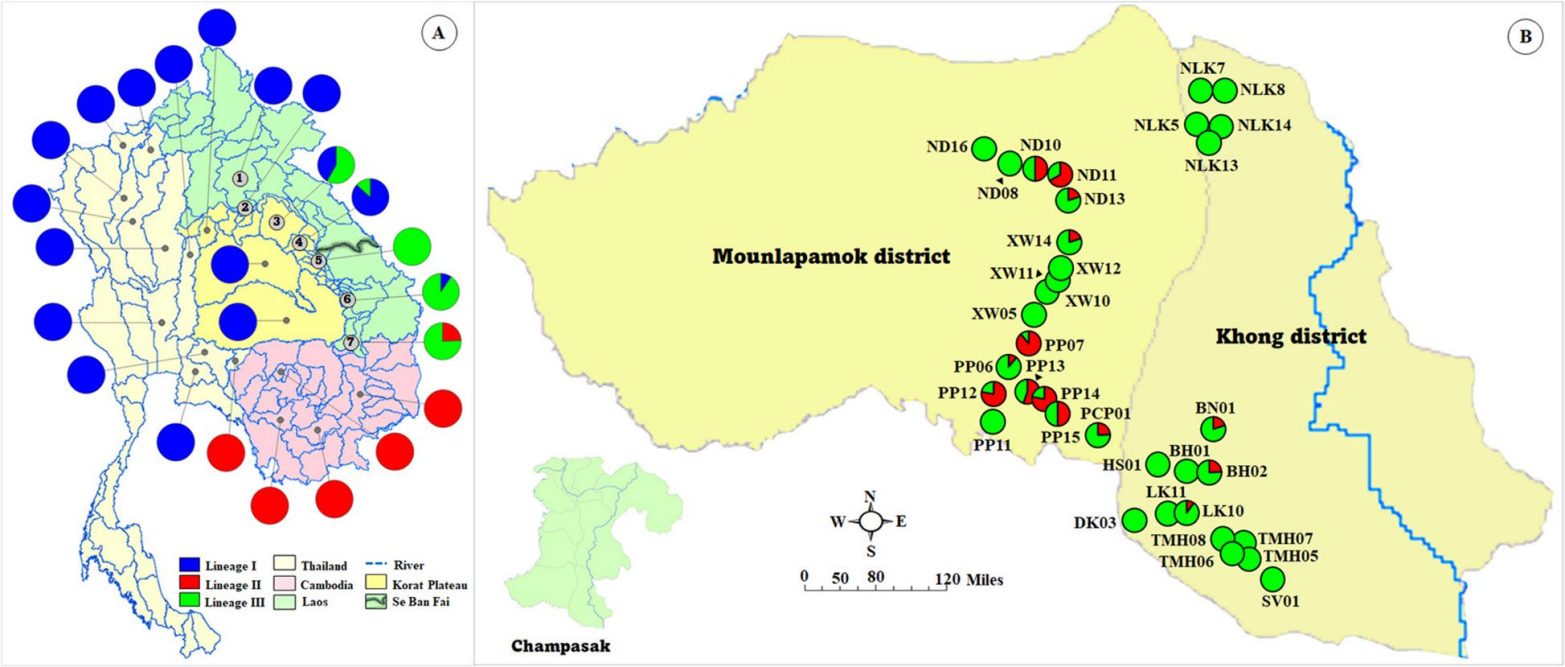
Distribution pattern of *Bithynia siamensis goniomphalos* within three (I–III) genetic lineages in the lower Mekong sub-region based on the *cox1* sequences: **A** distribution pattern in Thailand, Cambodia, and Laos. Catchments; 1; Huai Luang, 2; Nam Ngum, 3; Song Kram, 4; Nam Kam, 5; Se Bang Heang, 6; Se Bang Nouan, 7; Huai Tomo; **B** distribution pattern from Khong and Mounlapamok districts in Champasak Province: present study

This study represents the first DNA-based analysis of *Bithynia* spp. populations in Champasak Province, Laos, expanding molecular investigations of *B. s. goniomphalos*, which were previously confined to Vientiane and Savannakhet Provinces [5, 6]. Pairwise genetic differentiation (Φ_ST_) among snail populations from the 12 villages revealed that the non-significant genetic differentiation between Ban Na and Ban Huay was 0.003 based on the *cox1*, explaining snails can move between these villages. The presence of two distinct lineages (lineages II and III) of *B. s. goniomphalos* in the Champasak region. Lineage III was predominant, comprising 75.61% of the sampled population, while lineage II accounted for 24.39%. This distribution aligns with the geographic characteristics of Champasak Province, a southern Province located along the border of Laos and Cambodia, where lineage II has also been documented [5]. The coexistence of lineages II and III in Champasak highlights the complex population dynamics within this region. Lineage III appears to dominate the lower Mekong sub-region, with its distribution extending from Nakhon Phanom Province in Thailand through Savannakhet and Champasak Provinces in Laos (Fig. 4). The physical barriers restricting dispersal are affected by the snails moving. For example, lineage III, haplotype 127 was shared by 16 samples (three from Thailand and 13 from Laos), and haplotype 128 (H128) was shared by 20 samples (eight from Thailand and 12 from Laos) (Fig. 3). This data explained those countries are some geographically close and connected by flood plains. In addition, the Mekong and Tonle Sap rivers are the permanent water bodies, which are blocking the distribution of some species via these rivers.

However, populations of lineage II in southern Laos and along the Cambodian border were found to be genetically distinct, characterized by unique haplotypes and a divergence of 14 mutational steps between Lao and Cambodian populations (Fig. 3). Interestingly, the genetic structure among groups in those countries was significant differenced (*F*_CT_ = 0.660, *P* < 0.001, Table 5). These patterns suggest that the gene flow across these spatial scales is insufficient to counteract the effects of the genetic drift. The Li Phi (Somphamit) waterfall in Laos likely serves as a natural barrier [28], isolating the lineage II populations in Laos from those in Cambodia and Thailand. This natural barrier potentially limits gene flow between populations on either side of the waterfall and can fragment habitats and restrict the movement of species, leading to isolation. The isolation can reduce gene flow between populations, making them more susceptible to genetic drift. Indeed, the natural barrier, if it separates populations of a particular species or lineage, could prevent interbreeding between those populations. Over time, this lack of gene exchange might contribute to the divergence of genetic traits, as genetic drift becomes a more significant factor due to limited gene flow. The waterfall, with its strong current and difficult terrain, would likely make it challenging for individuals to cross and migrate between populations. This could explain why the populations in Laos (Lineage II) might be genetically distinct from those in Cambodia and Thailand, as the waterfall forms a physical boundary preventing or severely limiting gene flow. Such barriers often play a crucial role in speciation events, as they promote genetic isolation and potentially the development of new species over many generations.

The unique haplotypes between countries within lineage II can be explained by the lack of snail migration between those countries in lineage II. All individual samples were unambiguously assigned to their respective countries of origin, indicating minimal gene flow between populations across national borders. Interestingly, the ancestral lineage II may have originated in Laos, as the Lao populations exhibit the highest genetic diversity. This aligns with findings by Maes et al. [29], who reported that *Bulinus truncatus* snails in temporary habitats display greater genetic diversity than those in permanent habitats, despite a high self-fertilization rate in both environments. In Cambodia, lineage II populations predominantly inhabit temporary habitats and exhibit strong intra-population gene flow [5].

The genetic diversity and structure of *Bithynia* snail populations have significant implications for their susceptibility to *O. viverrini* infection. As evidenced in previous studies have demonstrated that genetic variation within snail populations such as *Bulinus truncatus*, *Biomphalaria glabrata*, and *Neotricula aperta* can influence their capacity to act as intermediate hosts for parasites [29–31]. In this study, the presence of three genetic lineages (*B. s. goniomphalos* lineages I, II, and III) across different geographic regions suggests that the varying genetic makeup of *Bithynia* populations could contribute to differential susceptibility to liver fluke infection. Lineages II and III, identified in southern Laos, exhibit overlapping distributions in several villages (e.g., Ban Huay, Ban Na, Phon Champa, and Longkang), creating populations with mixed genetic backgrounds. This genetic overlap may influence the susceptibility of these snail populations to liver fluke infections. A genetically diverse population, the range of immune responses and physiological traits among the snails would increase [30], which may affect their capacity to support the development of *O. viverrini* larvae. This means that not all snails are likely to be equally susceptible to parasites. Some individuals may have genetic traits that make them more resistant or capable of preventing infection, while others may have defenses that slow down the progress of the infection. On the other hand, genetically depauperate populations may lack sufficient diversity to mount effective resistance, potentially increasing infection prevalence [29].

Lineage II, predominantly found in Cambodia and parts of southern Laos inhabits temporary aquatic habitats with strong intra-population gene flow. Such environments may favor higher genetic homogeneity, which could enhance their suitability as intermediate hosts for *O. viverrini*. The higher genetic diversity observed in lineage II populations in Laos suggests a long evolutionary history and possibly a greater capacity for parasite–host adaptation, making these snails more susceptible to liver fluke infections (Bunchom et al. unpublic). The snail host and trematode parasite are co-evolution. Lineage II, predominantly found in Cambodia and parts of southern Laos inhabits temporary aquatic habitats with strong intra-population gene flow. Such environments may favor higher genetic homogeneity, which could enhance their suitability as intermediate hosts for *O. viverrini*. The higher genetic diversity observed in lineage II populations in Laos suggests a long evolutionary history and possibly a greater capacity for parasite–host adaptation, making these snails more susceptible to liver fluke infections. In contrast, lineage III, which dominates Laos, including Champasak Province, is associated with permanent aquatic habitats and exhibits a broader geographic range. The persistence of lineage III in these habitats might reduce the impact of genetic drift, maintaining sufficient genetic variation to resist or tolerate parasitic infections. However, its dominance in the lower Mekong sub-region, combined with the presence of mixed populations, may still facilitate the transmission cycle of *O. viverrini* by sustaining infections in snail hosts.

The relationship between genetic structure and parasite susceptibility is further complicated by environmental factors, such as water quality, habitat stability, and the presence of definitive hosts (e.g., humans, cats, and dogs) in the region. High infection prevalence in definitive hosts increases the likelihood of reinfection in snail populations, particularly in areas, where lineage II and III overlap. This overlap may enhance the transmission potential of *O. viverrini*, contributing to the high burden of opisthorchiasis in the region. Understanding the genetic basis of *Bithynia* snail susceptibility to *O. viverrini* is crucial for developing targeted control strategies. In natural habitats, the prevalence of *O. viverrini* infection in *B. s. goniomphalos* (8.37%) from Laos was higher than in Thailand (6.93%) [32]. In addition, *B. s. goniomphalos* is the highest susceptibility to *O. viverrini* [33], *B. s. siamensis* and *B. funiculata* remain potential hosts for transmission [34]. Molecular and ecological studies focusing on the interaction between *Bithynia* genetic lineages and liver fluke infection dynamics can help identify high-risk areas and inform interventions aimed at reducing transmission.

Interestingly, lineage I of *B. s. goniomphalos* was absent from the southern regions of Laos. This absence can likely be attributed to physical barriers, such as the Korat Basin in northeastern Thailand and the Se Bang Fai drainage in central Laos, which restrict dispersal [31]. Previous studies have primarily reported the presence of lineage I in northern and central Laos, further supporting the hypothesis of geographic isolation shaping its distribution. Moreover, only *B. s. goniomphalos* was identified in Laos, reinforcing the notion that lineage distribution in the region is heavily influenced by the flow of the Mekong and Tonle Sap rivers [5, 6].

This study confirms the existence of three distinct lineages of *B. s. goniomphalos* across Thailand, Laos, and Cambodia. These lineages are predominantly associated with stable highland streams and minor river systems rather than major rivers. For instance, *B. funiculata* is distributed along the Mekong Basin, likely originating from the Ping River valley, while *B. s. siamensis* is associated with the Mekong and Ping River systems, extending to the Gulf of Thailand via the Chao Phraya River [5, 6]. Genetic analyses reveal limited dispersal between local populations, which facilitates local adaptation to environmental conditions without significant genetic homogenization through gene flow [35].

The genus *Bithynia* exhibits far greater taxonomic diversity than previously recognized, underscoring the need for a comprehensive taxonomic revision [1–3]. Morphological differences, particularly in shell characteristics and ecological habitats, remain critical for diagnosing species complexes within the genus *Bithynia* [36]. However, traditional morphological approaches are insufficient for accurate identification without corroborative molecular data. Studies using molecular markers have revealed significant genetic divergence among the three lineages of *B. s. goniomphalos*, suggesting that ancestors of the genus *Bithynia* likely originated from mainland Southeast Asia [4–7].

## Conclusion

This study highlights the complex phylogeographic patterns and genetic diversity of *Bithynia* snails in Southeast Asia, emphasizing the need for integrative approaches to resolve their taxonomy and evolutionary history. The absence of lineage I in southern Laos, likely due to geographic barriers, such as the Korat Basin and Se Bang Fai drainage, underscores the role of physical and ecological factors in shaping lineage distributions. The coexistence of lineages II and III in southern Laos, with lineage III dominating in the lower Mekong sub-region, reflects the influence of major river systems like the Mekong and Tonle Sap on gene flow and population structure. The findings also reveal that the genus *Bithynia* exhibits significant taxonomic complexity, warranting a comprehensive revision using both morphological traits and molecular markers. Previous genetic studies indicate the non-monophyletic nature of Bithyniidae, necessitating the inclusion of highly polymorphic markers (e.g., arginine kinase introns) and broader sampling across Southeast Asia, particularly in Myanmar and Vietnam. Ultimately, understanding the genetic structure, lineage distribution, and taxonomy of *Bithynia* snails is critical for elucidating their role as intermediate hosts of *O. viverrini*. These insights will inform strategies for controlling liver fluke transmission and managing the ecological and evolutionary dynamics of snail–parasite interactions in the region.

## Data Availability

No datasets were generated or analysed during the current study.

## Abbreviations

*cox1*: Cytochrome *c* oxidase subunit 1
16S rRNA: 16S ribosomal RNA
*N*: Number of *B. s. goniomphalos* examined
*s*: Number of polymorphic sites
*H*: Number of haplotypes
*Uh*: Unique haplotypes
Hd: Haplotype diversity
NJ: Neighbor-joining
ML: Maximum likelihood
MJNs: Median-joining networks
WHO: World Health Organization
